# Feasibility of a Large Language Model Chatbot to Support Parental Understanding in the PICU

**DOI:** 10.1097/CCE.0000000000001378

**Published:** 2026-03-20

**Authors:** R. Brandon Hunter, Satid Thammasitboon, Sreya Rahman, Sriya Kakarla, Pamela Petersen, Shelley Kumar, Thomas Rappold, Sanjiv Mehta

**Affiliations:** 1 Department of Pediatrics, Baylor College of Medicine, Houston, TX.; 2 Division of Critical Care Medicine, Texas Children’s Hospital, Houston, TX.; 3 McGovern Medical School, Houston TX.; 4 Department of Anesthesiology and Critical Care Medicine, The Children’s Hospital of Philadelphia, Philadelphia, PA.; 5 Department of Pediatrics, Perelman School of Medicine, University of Pennsylvania, Philadelphia, PA.

**Keywords:** artificial intelligence, electronic health records, large language models, patient education, pediatric intensive care unit

## Abstract

**OBJECTIVES:**

To evaluate the feasibility of a large language model (LLM)-based chatbot for answering parental questions in the PICU and inform design of a randomized controlled trial (RCT).

**DESIGN:**

Prospective single-arm feasibility study conducted from August 2024 to December 2024.

**SETTING:**

Quaternary PICU.

**SUBJECTS:**

Fourteen parents of children admitted to the PICU.

**INTERVENTIONS:**

Parents engaged in 10-minute sessions with a HIPAA-compliant GPT-4o- (Generative Pretrained Transformer 4o, OpenAI, San Francisco, CA) based chatbot prompted with patient-specific electronic health record (EHR) data.

**MEASUREMENTS AND MAIN RESULTS:**

Feasibility was assessed through four criteria: parental engagement and satisfaction, provider perceptions, accuracy and safety, and recruitment. Of 16 eligible parents, 14 enrolled and completed all procedures (87.5% recruitment rate). Parents asked a median of six questions (range, 3–13) with 96% positive real-time satisfaction ratings. Post-interaction surveys demonstrated high perceived value (median, 5.0/6.0 across all domains; Net Promoter Score [NPS] +57). Of 1225 chatbot-generated sentences evaluated, 99.3% were accurate with all eight errors classified as minor (inter-rater reliability: Gwet’s AC2, a chance-corrected inter-rater agreement coefficient, = 0.98; 95% CI, 0.97–0.99). Healthcare providers rated response quality highly (median, 5.0/6.0), although physicians expressed greater comfort with bedside use of the tool than nurses (5.0 vs. 4.0; *p* = 0.004). Sample size calculations using NPS as the primary endpoint suggest enrolling 135 participants would provide adequate power for a future RCT.

**CONCLUSIONS:**

An EHR-informed LLM chatbot demonstrated high parental engagement and satisfaction, positive provider perception, and high accuracy and safety, supporting progression to a RCT.

**KEY POINTS**

**Question**: Can a HIPAA-compliant large language model (LLM) chatbot, prompted with patient-specific electronic health record (EHR) data, feasibly and accurately answer parental questions in the PICU?

**Findings**: In this single-arm feasibility study, parents demonstrated sustained engagement (median six questions per 10-min session, 96% positive real-time ratings) and high perceived value (Net Promoter Score +57). Healthcare providers rated response quality highly (median, 5.0/6.0). The chatbot achieved 99.3% accuracy with excellent inter-rater reliability (Gwet’s AC2 = 0.98) and no moderate or severe errors.

**Meaning**: This feasibility study supports progression to a randomized controlled trial using Net Promoter Score as the primary endpoint to evaluate whether EHR-informed LLM chatbots enhance parental understanding and communication in the PICU.

More than 240,000 children are admitted to U.S. PICUs annually, where parents must engage in high-stakes shared decision-making (1). Despite family-centered care initiatives like bedside rounds and open electronic health record (EHR) access, greater than 85% of PICU parents report persistent information gaps and frequent use of online search to supplement information from their medical teams (2, 3). However, traditional online resources lack patient-specific context and are typically written above recommended reading levels, leading these searches to double parental anxiety (3–5).

Large language models (LLMs) could address these gaps by providing accessible explanations tailored to parental questions and grounded in their child’s clinical data. We previously showed ChatGPT could incorporate patient-specific clinical data to deliver accurate, understandable responses to common PICU parental questions (6). However, real-world evaluation with actual patient data and parental interactions remains untested. Thus, we assessed feasibility of introducing a parent-facing HIPAA-compliant LLM chatbot to answer parental questions in the PICU.

## METHODS

### Design, Setting, and Patients

This single-arm prospective feasibility study was conducted from August 2024 to December 2024 in a quaternary PICU at a freestanding children’s hospital. The study was reviewed and approved by the Baylor College of Medicine Institutional Review Board (Protocol H-53797; approved July 10, 2023; study title: “Large Language Model Application in Healthcare: Use of Generative Pre-trained Transformer Models to Enhance Communication and Understanding in the Pediatric Hospital Setting”). Written informed consent was obtained from all participants before enrollment. All procedures were conducted in accordance with the ethical standards of the responsible institutional committee on human experimentation and with the Helsinki Declaration of 1975, as revised in 2013. This single-arm feasibility study was not registered as a clinical trial as it did not meet International Committee of Medical Journal Editors criteria for prospective registration, which require assignment of participants to health-related interventions for comparison. English-speaking parents who were comfortable using laptop computers were eligible. Primary attending physicians could exclude families at their discretion.

### Chatbot Platform and Study Procedures

Enrolled parents were offered a 10-minute unsupervised conversation with an artificial intelligence (AI) chatbot about their child’s PICU care. The chatbot was BastionGPT (FortaTech, LLC, Dallas, TX), a HIPAA-compliant GPT-4o-based platform (Generative Pretrained Transformer 4o, OpenAI, San Francisco, CA) with minimal healthcare fine-tuning. For each session, the principal investigator (PI) entered a system prompt (**Supplemental Table 1**, https://links.lww.com/CCX/B604) with chatbot instructions and patient-specific clinical data including most recent progress notes, laboratory and imaging results, and current medications.

Parents completed pre-interaction surveys (**Supplemental Table 2**, https://links.lww.com/CCX/B604), watched a 3-minute instructional video, and then engaged in unsupervised 10-minute conversations (**Supplemental Table 3**, https://links.lww.com/CCX/B604). Post-interaction, the PI reviewed transcripts with parents to address potential errors and administered a post-interaction survey. The patient’s ordering provider and bedside nurse separately reviewed transcripts for quality assessment.

### Outcome Measures and Statistical Analysis

Feasibility was evaluated through four criteria to support progression to a randomized controlled trial (RCT): 1) parental engagement and satisfaction, 2) provider perceptions, 3) accuracy and safety, and 4) recruitment.

Parental engagement was assessed by the number and frequency of questions, the type of question, and real-time satisfaction ratings (thumbs-up/down) given after each response. For a subset of conversations, detailed temporal data were extracted to assess sustained engagement throughout the session. Post-intervention user satisfaction and perceived value were measured using 6-point Likert scale questions (1 = “not at all” to 6 = “extremely”) assessing overall value, improved understanding, improved ability to communicate with healthcare team, ease of use, and understandability. We calculated the Net Promoter Score (NPS), a validated metric based on parents’ likelihood to recommend the chatbot on a 0–10 scale. NPS is calculated as the percentage of promoters (scores 9–10) minus detractors (scores 0–6), omitting passives (scores 7–8).

After reviewing conversation transcripts, the patient’s ordering provider and bedside nurse completed four-item surveys using 6-point Likert scales evaluating response quality, anticipated impact on parent understanding and patient care, and comfort with bedside use.

Sentence-level accuracy was assessed by the PI and ordering provider who evaluated transcripts separately without conferring using a 3-point severity scale: 1) minor errors unlikely to cause harm and easily clarified, 2) moderate errors likely to cause confusion, and 3) severe errors likely to cause patient or parent harm (**Supplemental Table 4**, https://links.lww.com/CCX/B604). Discrepancies were resolved by a third PICU physician. Inter-rater reliability was calculated using Gwet’s AC2 coefficient, which is a a chance-corrected inter-rater agreement coefficient that is less susceptible to prevalence effects than Cohen's kappa.

Continuous variables are presented as medians with interquartile ranges (IQRs). Categorical variables are presented as counts and percentages.

## RESULTS

### Participant Characteristics and Recruitment

Of 25 parents screened, 16 met eligibility criteria and 14 (87.5%) enrolled and completed the protocol. Parent baseline understanding and demographic characteristics as well as patient clinical variables are provided in **Supplemental Table 5** (https://links.lww.com/CCX/B604). Compared with Houston census data, our study population was representative with respect to education (36% bachelor’s degree or higher vs. 31%) and household income (36% below $50,000 vs. 35%), but had higher representation of non-Hispanic White families (~50% vs. 24%) with lower representation of Hispanic (36% vs. 45%) and Black (14% vs. 22%) families (7).

### Parent Engagement and Satisfaction

Parents demonstrated active engagement with the chatbot, asking a median of six questions (range, 3–13) during the 10-minute conversations. Ninety-six percent of rated responses (44/46) received thumbs-up (satisfactory) ratings. For a subset of conversations where detailed temporal data were extracted (*n* = 8), parents continued initiating questions until a median of 9.9 minutes (IQR, 7.6–10.0 min) into the session, with a median interquestion interval of 81 seconds (IQR, 58–120 s), reflecting continuous back-and-forth dialogue and engagement. Question content focused most commonly on therapeutic interventions (45%), diagnosis/monitoring (17%), and prognosis (17%) (**Supplemental Table 6**, https://links.lww.com/CCX/B604).

Post-interaction surveys using 6-point Likert scales (1 = “not at all” to 6 = “extremely”) demonstrated uniformly high ratings: overall value 5.0 (IQR, 5.0–5.0), improved understanding 5.0 (IQR, 4.0–6.0), improved ability to communicate with healthcare team 5.0 (IQR, 4.0–6.0), ease of use 6.0 (IQR, 5.0–6.0), and understandability 5.5 (IQR, 5.0–6.0; **Table 1**).

**TABLE 1.** Parent Satisfaction and Perceived Value of Chatbot Interaction

Interaction Evaluation (6-Point Likert^a^)	Median (IQR)
Overall value of the interaction	5.0 (5.0–5.0)
Improved understanding of child’s condition	5.0 (4.0–6.0)
Improved ability to communicate with healthcare team	5.0 (4.0–6.0)
Ease of interacting with the chatbot	6.0 (5.0–6.0)
Understandability of chatbot responses	5.5 (5.0–6.0)
Net Promoter Score	*n* (%)
Score	57
Promoters (9–10)	8 (57)
Passives (7–8)	6 (43)
Detractors (0–6)	0 (0)
Future Search Preference	*n* (%)
Prefer artificial intelligence chatbot	6 (42.9)
Equally use chatbot + current preferred source	6 (42.9)
Current preferred source	0 (0.0)
Did not answer	2 (14.3)

IQR = interquartile range.

a Likert scale items generally reflect that higher scores indicate better outcomes (e.g., greater satisfaction or perceived value).

This table presents parent responses regarding their satisfaction with and perceived value of the chatbot interaction. The Net Promoter Score and its breakdown into promoters, passives, and detractors are also reported. Data are summarized as medians (IQR) for Likert scale items and as *n* (%) for categorical responses.

The NPS was +57 (excellent), with 57% promoters and zero detractors. Among 12 parents who responded about their future information-seeking preferences if given continued chatbot access, 50% indicated they would prefer the AI chatbot exclusively and 50% would use both the chatbot and their current sources; no parents preferred traditional sources alone.

### Provider Perceptions

Both ordering providers and nurses rated overall response quality, impact on parent understanding, and impact on patient care highly (median, 5.0; IQR, 5.0–5.0 for all questions). Physicians expressed greater comfort with routine bedside use compared with nurses (median, 5.0 [IQR, 4.2–5.0] vs. 4.0 [IQR, 4.0–5.0]; *p* = 0.004 by Wilcoxon signed-rank test; **Table 2**).

**TABLE 2.** Healthcare Provider Perceptions of Chatbot Quality and Utility

Survey Item (1–6 Likert Scale)	Physician/Advanced Practice Provider, Median (IQR)	Bedside Nurse, Median (IQR)	*p*
Overall quality of chatbot responses	5.0 (5.0–5.0)	5.0 (5.0–5.0)	1
Impact on parent understanding	5.0 (5.0–5.0)	5.0 (5.0–5.0)	1
Impact on patient care	5.0 (5.0–5.0)	5.0 (5.0–5.0)	0.5
Comfort with parent use	5.0 (4.2–5.0)	4.0 (4.0–5.0)	0.004

IQR = interquartile range.

This table compares perceptions of chatbot response quality, anticipated impact on parental understanding and patient care, and comfort with use, between physicians/advanced practice providers and bedside nurses. Median scores (IQR) from a 6-point Likert scale are provided, along with statistical comparison results using the Wilcoxon signed-rank test.

### Accuracy and Safety Assessment

Of 1225 chatbot-generated sentences, 1217 (99.3%) were ultimately classified as accurate and 8 (0.7%) as errors. Thirty sentences (2.4%) required adjudication by a third PICU physician to resolve initial disagreements between reviewers; 22 of these were judged accurate and eight as errors. All eight errors (0.7%) were classified as minor; no moderate or severe errors were identified. Qualitative analysis of these errors revealed inaccuracies in clinical nuance, medication indication, and inappropriate reporting of medical details. Inter-rater reliability was excellent (Gwet’s AC2 = 0.98; 95% CI, 0.97–0.99).

## DISCUSSION

This feasibility study demonstrates that a HIPAA-compliant LLM chatbot can effectively answer parental questions in the PICU, supporting progression to a RCT. Four feasibility criteria were met: 1) high parental engagement and reported value (median satisfaction ≥ 5.0/6.0 across all questions, NPS +57), 2) positive bedside provider perception (median quality ratings 5.0/6.0), 3) high accuracy and safety (99.3% accuracy with no severe errors), and 4) adequate recruitment (87.5% enrollment rate).

To inform a RCT, we selected the NPS as the primary endpoint to capture the overall value and likelihood of adoption by families. Sample size calculations for an RCT require assumptions about control group performance, but baseline NPS data for standard online health resources are limited. Therefore, we used a reported System Usability Scale (SUS) score of approximately 70 for WebMD to represent baseline performance of standard online health resources (8). Using validated SUS-to-NPS conversion models, we estimated this corresponds to an NPS of approximately 0, allowing us to power the trial to detect a 30-point NPS difference between chatbot and control groups (9, 10). For the pilot cohort, individual recommendation ratings (0–10) yielded a mean NPS-coded score of +57 with an estimated sd of about 51 when coded as –100 (detractors), 0 (passives), and +100 (promoters). To allow for greater heterogeneity in a larger trial, we assumed a conservative sd of 55. Under these assumptions, a two-sided α of 0.05 and 80% power yield a required sample size of 54 participants per arm. Allowing for approximately 20% attrition, we plan to enroll about 135 parents.

This single-center feasibility study has important limitations. The quaternary care setting may not reflect community PICU experiences. The 10-minute interaction limit may not capture sustained use patterns. Manual EHR data entry limits scalability. English-only enrollment and documented racial and ethnic disparities in LLM adoption may have contributed to underrepresentation of Hispanic and Black families in our cohort, limiting generalizability (11, 12). Loss of real-time satisfaction (only 46 responses with recovered ratings) and temporal engagement data (available for 8/14 conversations) due to the platform’s HIPAA-compliant 30-day automatic data deletion policy limit our ability to fully characterize user engagement patterns. While pilot data demonstrate feasibility and acceptability, a definitive RCT would benefit from more objective endpoints including observed communication quality, validated knowledge and anxiety assessments, and documented information-seeking behaviors. Sample size calculations relied on assumptions about control group NPS and variance given limited published benchmarks for standard online health resources.

A RCT is needed to determine whether this technology meaningfully enhances parental understanding and communication in the PICU. Essential technical and safety enhancements for an RCT would include automated EHR integration via fast healthcare interoperability resources (FHIR) application programming interfaces (APIs) to eliminate manual data entry, automated content monitoring to flag potentially harmful responses, explicit disclaimers guiding parents to contact their team directly for urgent concerns, and daily check-ins with periodic reviews of flagged conversations by study personnel. We envision the chatbot as a primarily patient-facing intervention that will serve as a supplement to rather than a substitute for bedside communication. Parents would use chatbot responses to learn and formulate more informed questions during rounds, facilitating more productive clinician dialogue. Implementation studies should evaluate whether chatbot access enhances the quality of parent-clinician interactions without creating false reassurance or reducing direct communication with the medical team.

## Supplementary Material



## References

[1] Killien EY Keller MR Watson RS: Epidemiology of intensive care admissions for children in the US from 2001 to 2019. JAMA Pediatr 2023; 177:506–515 36972043 10.1001/jamapediatrics.2023.0184 PMC10043802

[2] Yudianto B Caldwell PH Nanan R: Patterns of parental online health information-seeking behaviour. J Paediatr Child Health 2023; 59:743–752 37051735 10.1111/jpc.16387

[3] Kubb C Foran HM: Online health information seeking by parents for their children: Systematic review and agenda for further research. J Med Internet Res 2020; 22:e19985 32840484 10.2196/19985 PMC7479585

[4] Hutchinson N Baird GL Garg M: Examining the reading level of internet medical information for common internal medicine diagnoses. Am J Med 2016; 129:637–639 26829438 10.1016/j.amjmed.2016.01.008

[5] Rooney MK Santiago G Perni S: Readability of patient education materials from high-impact medical journals: A 20-year analysis. J Patient Exp 2021; 8:2374373521998847 34179407 10.1177/2374373521998847 PMC8205335

[6] Hunter RB Thammasitboon S Rahman SS: Using ChatGPT to provide patient-specific answers to parental questions in the PICU. Pediatrics 2024; 154:e2024066615 39370900 10.1542/peds.2024-066615

[7] Census Reporter: Census Profile: Houston, TX. 2023. Available at: http://censusreporter.org/profiles/16000US4835000-houston-tx/. Accessed December 31, 2025

[8] Alduhailan HW Alshamari MA Wahsheh HAM: A comprehensive comparison and evaluation of AI-powered healthcare mobile applications’ usability. Healthcare (Basel) 2025; 13:1829 40805861 10.3390/healthcare13151829 PMC12345953

[9] Sauro J Lewis JR: Quantifying the User Experience: Practical Statistics for User Research. Second Edition. Morgan Kaufmann, Waltham, MA, 2016

[10] Sauro J: A Practical Guide to the System Usability Scale: Background, Benchmarks & Best Practices. Denver, CO, Measuring Usability, LLC, 2011

[11] Gelles-Watnick R Park E: Most Americans Haven’t Used ChatGPT; Few Think It Will Have a Major Impact on Their Job. Pew Research Center. 2023. Available at: https://www.pewresearch.org/short-reads/2023/08/28/most-americans-havent-used-chatgpt-few-think-it-will-have-a-major-impact-on-their-job/. Accessed October 16, 2024

[12] Sidoti O Park E Gottfried J: About a Quarter of U.S. Teens Have Used ChatGPT for Schoolwork—Double the Share in 2023. Pew Research Center. 2025. Available at: https://www.pewresearch.org/short-reads/2025/01/15/about-a-quarter-of-us-teens-have-used-chatgpt-for-schoolwork-double-the-share-in-2023/. Accessed March 6, 2025

