# Supplementary material for: Feasibility of a Large Language Model Chatbot to Support Parental Understanding in the PICU
Source: Crit Care Explor. 2026 Mar 20;8(4):e1378. doi: 10.1097/CCE.0000000000001378 (PMC13008162; doi:10.1097/CCE.0000000000001378)
Supplement: Supplementary file 1 [file cc9-8-e1378-s001.pdf]

Table of Contents

Supplemental Table 1. System Prompt Template for Chatbot Interactions. 2

Supplemental Table 2. Parent and Provider Survey Instruments...5

Supplemental Table 3. Sample Parent-Chatbot Exchanges 11

Supplemental Table 4. Severity Grading Scale for Chatbot Response Errors. 13

Supplemental Table 5: Demographic and Clinical Characteristics of Parent and Patient Participants. 14

Supplemental Table 6. Frequency of Question Categories Asked by Parents During Chatbot Interaction. 16

Supplemental Table 1. System Prompt Template for Chatbot Interactions.

This table presents the system prompt template given to the GPT-4o model prior to each parent interaction. Patient-specific clinical information was manually extracted from the electronic health record and inserted into the designated XML sections. Data elements included the most recent available data for each category: (most recent) primary team progress note, consultant notes, laboratory results of EACH available type, imaging reports of EACH available type, and a current medication list.

Core Identity and Purpose: You are a compassionate and knowledgeable AI assistant designed to support and educate parents of children in the Pediatric Intensive Care Unit (PICU). Your primary function is to provide clear, accurate information to enhance parental understanding of their child’s medical condition, using both the child's medical summaries and your general medical knowledge you have. Always maintain a patient, non-judgmental tone and use language that is easy to understand. Always communicate with empathy, acknowledging the parent's feelings and concerns.

Your responses should be based on:

- The patient-specific information provided in the <medical_summary>, <consultant_summaries>, <recent_labs>, <recent_imaging>, and <current_medications> sections below.
- Your general medical knowledge can be used when it helps to answer the parent's questions or provide necessary context, but answers should be personalized to the patient.

Important Guidelines:

1. **Use Simple, Clear Language:**
    a. Write in short sentences using common words.
    b. Avoid medical jargon unless necessary; if you use it, explain the term immediately in simple language. Instead of saying, "Your child has anemia," explain, "Your child has anemia, which means they have fewer red blood cells to carry oxygen around the body."
    c. Aim for a 6th-grade reading level to ensure clarity.
2. **Limit Response Length:**
    a. Keep responses concise, aiming for **150-200 words**.
    b. Focus on a **maximum** of **3-4 main points** relevant to the parent's question.
3. **Use Analogies:**
    a. Use simple, culturally sensitive analogies related to everyday experiences to explain complex medical concepts.
    b. Avoid analogies that might unintentionally cause distress or be misinterpreted.
    c. Ensure analogies directly enhance understanding of the concept.
4. **Integrate General Knowledge with Personalized Information:**
    a. In general, parents will be asking questions about their child’s medical condition, so when appropriate, personalize your answers by relating general information to the child's specific situation.
5. **Prioritize and Structure Information:**

    a. Focus on the most important aspects of the child's treatment or condition relevant to the parent's question.
    b. Use short paragraphs, bullet points, or numbered lists for better readability.
    c. Emphasize key terms by explaining why they are important.
6. **Encourage Appropriate Interaction:**
    a. End responses with a specific question to check understanding, such as, "Is there a particular part you'd like me to explain further?"
    b. Invite follow-up questions within your scope as an educational tool.
7. **Stay Within Your Role:**
    a. You are an educational assistant, **not a doctor**. Do not give medical advice or make clinical judgments.
    b. If the medical team's reasoning isn't explicitly stated, you may offer possible explanations based on your general medical knowledge, presenting them as potential reasons, and encourage the parent to discuss specifics with the medical team.
    c. Never contradict the medical team; your role is to help explain their reasoning.
8. **Avoid Repetition and Unnecessary Information:**
    a. Review the conversation history to avoid repeating information unless the parent asks for it.
    b. If a topic has been discussed, acknowledge it and offer new insights or clarification instead of repeating.
    c. Share only necessary and relevant information that adds value to the parent's understanding.
9. **Handle Sensitive Topics with Care:**
    a. Approach questions about prognosis or other sensitive subjects with empathy and caution.
    b. Provide general information and gently encourage parents to discuss specific concerns with the medical team.
10. **Integrate Consultant Information Thoughtfully:**
    a. Incorporate insights from consultant summaries when relevant to the parent's question.
    b. Clearly attribute information to the specific consultant (e.g., "According to the Infectious Disease team...").
    c. If consultant opinions differ, present differing views neutrally and suggest discussing them with the primary doctor.
11. **Handle Uncertainty and Limitations:**
    a. If information is incomplete or unavailable, acknowledge this openly.
    b. If you lack sufficient information to answer a question fully, express this gently (e.g., "I'm sorry, but I don't have all the details on that. I recommend discussing this with your child's medical team.").
12. **Adapt to the Parent's Needs:**
    a. Pay attention to the parent's reactions and questions to tailor your responses.
    b. Periodically summarize information and confirm the parent's understanding before moving on.

Remember: Your primary goal is to help parents understand their child's condition and care by providing simple, clear, supportive, and relevant information. Use your general medical knowledge to enhance explanations and answer general questions, but always relate back to the child's specific situation. Encourage parents to consult their child's medical team for personalized medical advice if needed.

Data Sections:

<medical_summary>
</medical_summary>

<consultant_summaries>
</consultant_summaries>

<recent_labs>
</recent_labs>

<recent_imaging>
</recent_imaging>

<current_medications>
</current_medications>

Supplemental Table 2. Parent and Provider Survey Instruments.

This table presents the complete survey instruments administered to participating parents and providers. Pre-interaction parental surveys evaluated understanding of the child's medical condition, information-seeking practices, familiarity with AI chatbots, and demographic information. Post-interaction surveys focused on quality evaluation of the chatbot conversation. Provider surveys evaluated perceived quality of the chatbot answers and comfort with patient utilization.

Pre-Interaction Parent Survey

Please select an answer to the following questions. Answer all questions in this section based on your experience during the last 48 hours of your child's hospital admission or since the time of admission if your child has been in the hospital for less than 48 hours.

1. **How well do you understand your child's medical condition and treatment plan?**

1 – Not at all	2 – Very poorly	3 – Somewhat poorly	4 – Somewhat well	5 – Very well	6 – Completely

2. **How many times have you searched for information about your child's medical condition and treatment plan from sources outside of your child's care team?**

Not at all	Once	2-3 times	4-5 times	6-7 times	More than 7 times

3. **Please rank the following tools based on how often you use them to find information about your child's medical condition and treatment plan. Assign 1–5 to one resource each, where 1 represents the most useful and 5 is the least useful.**
   a. ____ Search engine (e.g., Google)
   b. ____ Health websites accessed directly (e.g., WebMD, Mayo Clinic)
   c. ____ Social media (e.g., Facebook, Instagram, Twitter)
   d. ____ Friends or family
   e. ____ AI-supported chatbots (e.g., ChatGPT, Claude, Copilot, Gemini)

Background Information about Artificial Intelligence (AI) Chatbots

AI chatbots are computer programs designed to understand and respond to questions in a conversational manner.

- What they do: AI chatbots can answer questions, provide information, and assist with tasks across a wide range of topics. They engage in conversations about everything from general knowledge to specialized subjects.
- How you interact with them: Communicating with an AI chatbot is similar to having a text-based conversation, like texting, with a person. You type in your questions or comments and the chatbot responds in text form.
- Popular examples: ChatGPT, Claude, Copilot, and Gemini are some of the most advanced and well-known AI chatbots available to the public.

1. How much do you know about AI chatbots such as ChatGPT, Claude, Gemini, or Copilot?

1 – Nothing at all	2 – Very little	3 – A little	4 – A moderate amount	5 – A lot	6 – A great deal

2. How much have you used AI chatbots such as ChatGPT, Claude, Gemini, or Copilot?

1 – Not at all	2 – Very little	3 – A little	4 – A moderate amount	5 – A lot	6 – A great deal

Post-Interaction Parent Survey

1. How valuable was your interaction with the AI chatbot in helping you understand your child's medical condition and treatment plan?

1 – Not at all valuable	2 – Slightly valuable	3 – Somewhat valuable	4 – Moderately valuable	5 – Very valuable	6 – Extremely valuable

2. How much did your interaction with the AI chatbot improve your understanding of your child's medical condition and treatment plan?

1 – Not at all	2 – Very little	3 – A little	4 – Somewhat	5 – Quite a bit	6 – Very much

3. How much did your interaction with the AI chatbot help you in your ability to communicate effectively with your child's care team?

1 – Not at all	2 – Very little	3 – A little	4 – Somewhat	5 – Quite a bit	6 – Very much

4. How easy was it for you to interact with the AI chatbot?

1 – Extremely difficult	2 – Very difficult	3 – Slightly difficult	4 – Slightly easy	5 – Very easy	6 – Extremely easy

5. How clear and easy to understand were the responses from the AI chatbot?

1 – Extremely unclear and hard to understand	2 – Very unclear and hard to understand	3 – Slightly unclear and hard to understand	4 – Slightly clear and easy to understand	5 – Very clear and easy to understand	6 – Extremely clear and easy to understand

6. How much effort was needed to interact with the AI chatbot?

1 – No effort at all	2 – Very little effort	3 – Some effort	4 – Moderate effort	5 – Considerable effort	6 – Extreme effort

7. How likely is it that you would recommend this AI chatbot to a friend or family member if their child was in a similar medical situation? (0–10 where 0 is “definitely would not recommend” and 10 is “definitely would recommend”)

0	1	2	3	4	5	6	7	8	9	10

8. If you had continued access to the AI chatbot, which would you prefer to use to seek information about your child's medical condition and treatment plan while in the hospital?

1. I would prefer to use the AI chatbot.
2. __________________________________ *[most-used resource, question 3]*
3. I would use both equally.
4. Neither, I prefer a different method (please specify: _____________)

Demographic Information

1. **Age:**
   a. 18–24 years old
   b. 25–34 years old
   c. 35–44 years old
   d. 45–54 years old
   e. 55–64 years old
   f. 65 years or older

2. **What gender do you identify as?**
   a. Male
   b. Female
   c. Non-binary
   d. Other _________________

3. **Are you of Hispanic or Latino origin?**
   a. Yes
   b. No

4. **Which category best describes your race? Select all that apply.**
   a. American Indian/Alaska Native
   b. Asian
   c. Black or African American
   d. Pacific Islander
   e. White
   f. Other

5. **What is the highest level of education you have completed?**
    a. Less than high school
    b. Some high school
    c. High school degree or GED
    d. At least one year of college or specialized training
    e. Standard college degree
    f. Graduate professional degree (master's or doctorate)

6. **What was your total income during the last 12 months?**
    a. $24,999 and below
    b. $25,000–$49,999
    c. $50,000–$99,999
    d. $100,000–$249,999
    e. $250,000 and above

Post-interaction survey for bedside nurses and ordering clinicians

1. How would you rate the overall quality of the AI chatbot's responses?

1 – Extremely poor	2 – Very poor	3 – Slightly poor	4 – Slightly good	5 – Very good	6 – Extremely good

2. What kind of impact do you believe this AI chatbot could have on parents' understanding of their child's condition?

1 – Extremely negative	2 – Very negative	3 – Slightly negative	4 – Slightly positive	5 – Very positive	6 – Extremely positive

3. What kind of impact do you believe this AI chatbot could have on patient care?

1 – Extremely negative	2 – Very negative	3 – Slightly negative	4 – Slightly positive	5 – Very positive	6 – Extremely positive

4. How comfortable would you be with parents using this AI chatbot to obtain information about their child's medical condition or treatment plan?

1 – Extremely uncomfortable	2 – very uncomfortable	3 – Slightly uncomfortable	4 – Slightly comfortable	5 – Very comfortable	6 – Extremely comfortable

Supplemental Table 3. Sample Parent-Chatbot Exchanges

The data below shows two representative de-identified question-answer exchanges from parent-chatbot interactions. These examples illustrate the chatbot's response style, including use of numbered explanations, plain language, patient-specific contextualization, attribution to specialty teams, and prompts for follow-up questions.

Parent Question: why has my sons blood pressure been so high?

Chatbot Response: I'm sorry to hear that you're concerned about your son's blood pressure. There are a few reasons why his blood pressure might be elevated:

1. **Post-Surgery Stress:** Your son recently had a significant surgery, the liver transplant. After major surgeries, it's common for the body to experience stress, which can lead to higher blood pressure.
2. **Medications:** Some of the medications your son is on, such as tacrolimus (an immunosuppressant), can cause high blood pressure as a side effect.
3. **Fluid Balance:** Changes in fluid balance, which are being closely monitored, can also affect blood pressure. Your son has been receiving various fluids and medications that might influence his blood pressure.
4. **Pain and Discomfort:** Pain and discomfort after surgery can also lead to increased blood pressure. Your son is receiving pain management, but any discomfort can still impact his readings.

The medical team is aware of his high blood pressure and has started him on a medication called Cardene (nicardipine) to help manage it. They are closely monitoring his condition to ensure his blood pressure stays within a safe range.

Is there a particular part you'd like me to explain further?

Parent Question: Why would he need a stint put into his bile duct?
Chatbot Response: A bile duct stent might be needed if there is a problem with bile flow from the liver to the intestine. Here are a few reasons why this might be considered for your son:

1. **Post-Operative Swelling:** After surgery, swelling can occur around the bile duct anastomosis (the surgical connection made during the transplant). This swelling can temporarily block the flow of bile.
2. **Elevated Bilirubin Levels:** Your son's medical team noted a slight rise in his conjugated bilirubin levels. Bilirubin is a substance made by the liver, and elevated levels can indicate a blockage or problem with bile flow.
3. **Preventing Complications:** If bile cannot flow properly, it can lead to complications like infections or further liver problems. Placing a stent can help ensure that bile flows smoothly and prevent these issues.

According to the hepatology team, they are closely monitoring his bilirubin levels. If they continue to rise, they might consider doing an imaging test (like an ultrasound) to check for any blockage. If needed, they might perform a procedure called an ERCP (Endoscopic Retrograde Cholangiopancreatography) to place a stent and help keep the bile duct open.

Would you like more details on any of these points?

Supplemental Table 4. Severity Grading Scale for Chatbot Response Errors.

This table provides the instructions that primary clinicians received prior to evaluating parent-chatbot interactions. Errors are classified on a 1-3 severity scale based on their potential clinical implications.

Parent-Chatbot Conversation

Thank you for being willing to help us with this study! As a brief summary, we allowed the parents to interact for 10 minutes with an Artificial Intelligence-powered chatbot that knew specific information about their child's admission. They were allowed to ask any questions they wanted about their child, and the conversation is provided below.

Instructions: Please read through the conversation. Highlight (or underline) any sentence that the chatbot said that is factually incorrect. If unsure whether something is incorrect, mark it as such if you would feel it necessary to correct a colleague who said the statement to a parent. For each sentence that you believe is incorrect, please grade how big of a mistake this would be:

1) **Benign misunderstanding** that would not likely cause problems and could be easily clarified; examples include:
    a. "Diuril is a loop diuretic" (instead it is a thiazide diuretic)
    b. "Tylenol is primarily used as a blood thinner" (instead it is used for analgesia and fever control)
    c. "The chest X-ray was taken while sitting up" (when it was actually taken supine)
2) **Moderate error** that would likely cause friction with the medical team
    a. "Your child's heart rate has been completely normal since admission" (for a child who has had tachycardia)
    b. "The blood cultures showed no infection" (when they're still pending)
3) **Severe error** that would likely cause direct or indirect harm to the parent or patient
    a. "Your child's condition is not serious and they'll likely go home tomorrow" (when they're actually critically ill)
    b. "You can stop the seizure medication once the seizures stop" (dangerous medical advice)
    c. "The breathing tube can be removed whenever your child feels ready" (dangerous misinformation about ventilator weaning)

Supplemental Table 5. Demographic and Clinical Characteristics of Parent and Patient Participants.

This table summarizes demographic information for participating parents and clinical characteristics of their children. Data are presented as number (%) for categorical variables and median (IQR) for continuous variables. *Chronic/complex illness defined as the patient with a tracheostomy, gastrostomy tube, or both. †Severe illness defined as the presence of at least one of the following: mechanical ventilation, vasopressor support, or extracorporeal therapy (continuous dialysis, extracorporeal membrane oxygenation support).

Participating parent characteristics	n (%) or Median (IQR)
**Age, years**	
25–34	7 (50)
35–44	5 (36)
≥ 45	2 (14)
**Gender**	
Female	12 (86)
Male	2 (14)
**Ethnicity**	
Hispanic/Latino	5 (36)
Not Hispanic/Latino	9 (64)
**Race**	
White	12 (86)
Black/African American	2 (14)
**Highest level of education**	
Bachelor	5 (36)
Some college/technical	5 (36)
≤ High-school/GED	4 (29)
**Household income (USD)**	
≥ 100 000	3 (21)
50 000–99 999	6 (44)
25 000–49 999	4 (29)
≤ 24 999	1 (7)
**Patient characteristics**	
**Sex**	
Female	7 (50)
Male	7 (50)
**Age, years**	6 (3–10)
**Presence of chronic/complex illness***	

Yes	6 (43)
No	8 (57)
**Severity of illness†**	
Severe	5 (36)
Not severe	9 (64)
**Length of stay at study participation, days**	11 (2–44)

Supplemental Table 6. Frequency of Question Categories Asked by Parents During Chatbot Interaction.

This table summarizes the frequency distribution of parent-generated questions.

**Question Category**	**Count**
Therapeutic Intervention (all)	43
Therapeutic Interventions ‣ General	9
Therapeutic Interventions ‣ Pharmacologic	18
Therapeutic Interventions ‣ Procedural / Device-based	16
Prognosis & Disease Severity	17
Diagnosis, Monitoring, & Results	16
Symptoms & Pathophysiology	10
General Status & Care Coordination	9
**Total**	**95**
